# 尿液细胞外囊泡的高效亲和纯化捕获与蛋白质组学分析

**DOI:** 10.3724/SP.J.1123.2024.11013

**Published:** 2025-05-08

**Authors:** Guiyuan ZHANG, Zhen ZHAN, Weiguo TAO, Hao ZHANG

**Affiliations:** 1.东南大学生物科学与医学工程学院, 江苏 南京 210096; 1. School of Biological Science & Medical Engineering, Southeast University, Nanjing 210096, China; 2.逸微健华生物科技有限公司, 江苏 南京 210032; 2. EVLiXiR Biotech, Nanjing 210032, China; 3.南京紫金山分子医学技术研究院, 江苏 南京 210032; 3. Bell Mountain Molecular MedTech Institute, Nanjing 210032, China; 4.普渡大学生物化学系, 印第安纳州 西拉法叶 47907; 4. Department of Biochemistry, Purdue University, West Lafayette, IN 47907, America

**Keywords:** 细胞外囊泡, 尿液, 蛋白质组学, 前列腺癌, 亲和纯化, extracellular vesicles, urine, proteomics, prostate cancer, affinity purification

## Abstract

目前,液体活检因其创伤小、易获取且操作简便的优点,已成为癌症诊断中传统组织活检的替代方法。尿液来源的细胞外囊泡(extracellular vesicles, EVs)已被确定为癌症生物标志物的重要来源。EVs是由细胞分泌的磷脂双分子层囊泡,内含蛋白质、DNA及RNA等成分,且由于双层磷脂膜的保护,EVs内的蛋白质可免受体液中酶的降解。然而,在蛋白质组学分析中,低产率使得从生物体液中分离EVs仍是一个挑战。在本研究中,我们采用亲和磁珠EVlent对尿液中的EVs进行富集,EVlent通过识别EVs表面的特异性蛋白质实现高选择性富集。首先使用蛋白免疫印迹、纳米粒子追踪分析和透射电子显微镜对尿液中的EVs进行综合表征,结果表明,EVlent亲和磁珠成功的从尿液中分离出了EVs,且富集效果优于传统的超速离心方法。接下来,将此方法应用于15例健康志愿者和15例前列腺癌患者的尿液EVs蛋白质组学分析,并筛选潜在的肿瘤标志物。结果显示,健康对照组平均鉴定出2039种蛋白质和14490条肽段,前列腺癌患者中分别鉴定出1982种蛋白质和13100条肽段。进一步分析发现,91种蛋白质属于Vesiclepedia数据库中最常见的100种EV蛋白质。与健康对照组相比,前列腺癌患者尿液EVs中88种蛋白质表达上调,90种蛋白质下调。KEGG分析揭示4种蛋白质(尿激酶型纤溶酶原激活剂(PLAU)、血小板衍生生长因子A(PDGFA)、基质金属蛋白酶3 (MMP3)和神经母细胞瘤RAS病毒癌基因同源物(NRAS))在前列腺癌通路中富集,这些蛋白质未来有希望作为前列腺癌的潜在生物标志物,可以为前列腺癌的早期诊断及预后提供重要依据。

## Abstract (en)

Liquid biopsy is a promising alternative to traditional tissue biopsies for diagnosing cancer because it offers advantages such as minimal invasiveness, accessibility, and ease of operation. Extracellular vesicles (EVs) are lipid bilayer vesicles that contain proteins, DNA, and RNA and are secreted by cells. Indeed, urinary EVs are important sources of cancer biomarkers. The lipid bilayer protects EV proteins from degradation by enzymes present in bodily fluids. Prostate cancer (PCa) is among the most prevalent malignancies in developed countries and is the second-leading cause of cancer-related mortality in men. Current screening methods commonly used to initially evaluate patients with suspected PCa include serum prostate-specific antigen (PSA) testing and digital rectal examination (DRE), with magnetic resonance imaging (MRI) and transrectal ultrasound often recommended for further assessment. However, both PSA testing and DRE have limited specificities, which results in a substantial number of unnecessary prostate biopsies. Consequently, additional reliable biomarkers need to be urgently discovered for rapidly diagnosing PCa more accurately. Prostate-derived secretions, including those associated with malignancies, are detectable in urine owing to the anatomical proximity of the prostate to the urethra; hence urine is a promising liquid-biopsy medium for discovering PCa biomarkers, which is a topic that has been the focus of extensive research efforts in recent years. However, isolating EVs from biofluids in sufficient yields for proteomics analysis remains challenging.

In this study, functional magnetic beads EVlent (extracellular vesicles isoLated efficiently, naturally, and totally) with high-affinity capabilities were developed for selectively enriching EVs from biological fluids.The surfaces of the beads were modified with three antibodies that target CD9, CD63, and CD81, which enables the specific recognition of EV surface proteins. The isolation performance of EVlent was validated by comprehensively characterizing urinary EVs using Western blotting (WB), nanoparticle tracking analysis (NTA), and transmission electron microscopy (TEM). WB revealed prominent bands for EV markers (CD9, TSG101, and HSP70) in EVlent-enriched samples, whereas weaker bands were observed following ultracentrifugation (UC). NTA revealed that the EVs isolated by EVlent are predominantly in the 50-400 nm size range, with a content of 4.1×10^9^ particles/mL, which is significantly higher than the value of 1.8×10^9^ particles/mL obtained by UC. TEM confirmed that the isolated EVs have characteristic elliptical or cup-shaped vesicular structures. These findings demonstrate that EVlent outperforms UC in terms of enrichment efficiency and purity, delivering a separation efficiency of 87.2% compared to the value of 30.3% obtained by UC. We used proteomics to analyze urinary EVs isolated from 15 healthy volunteers and 15 patients with prostate cancer using EVlent affinity magnetic beads with the aim of identifying potential biomarkers for prostate cancer. On average, 2039 proteins and 14490 peptides were identified in the control group, while 1982 proteins and 13100 peptides were identified in the patient group. Further analysis revealed 91 proteins commonly found in the Vesiclepedia database (Top 100). Compared with the healthy volunteers, 88 proteins were upregulated and 90 proteins were downregulated in patients with prostate cancer. Gene ontology (GO) analysis showed that these upregulated proteins are enriched in extracellular exosomes, extracellular space, extracellular region, collagen-containing extracellular matrix, proteolysis and protein-binding. Pathway analysis using the Kyoto Encyclopedia of Genes and Genomes (KEGG) highlighted associations between ribosomes, protein digestion and absorption, complement and coagulation cascades, prostate cancer, transcriptional misregulation in cancer, aldosterone-regulated sodium reabsorption, endocrine and other factor-regulated calcium reabsorption, and pancreatic secretion. Notably, four proteins including plasminogen activator urokinase (PLAU), platelet-derived growth factor subunit A (PDGFA), matrix metalloproteinase 3 (MMP3), and neuroblastoma RAS viral oncogene homolog (NRAS) were identified within the prostate cancer pathway, highlighting their potential as biomarkers for the early diagnosis and prognosis of prostate cancer. In conclusion, this study introduced EVlent as a robust platform for the efficient isolation and proteomics analysis of EVs, providing valuable insight into urinary EV biomarkers and their clinical prostate-cancer applications.

胞外囊泡(extracellular vesicle, EVs)是一类由脂质双分子膜包裹的膜性囊泡,内含多种蛋白质、脂质及核酸。胞外囊泡通过质膜的外向出芽或内体膜的内向出芽从起源细胞释放,形成多泡体并在与质膜融合时释放其内容物。囊泡的释放促进细胞间通讯,既可以通过与内容物接触,也可以通过质膜融合或内吞作用进入“受体”细胞^[1⇓⇓-4]^。最初,EVs的释放被认为是细胞排除多余或无用的物质,然而后续研究表明,EVs在细胞间通讯中扮演着重要角色,参与正常生理过程及病理进展^[5⇓-7]^。根据分泌方式或尺寸,EVs目前被分类为不同类型。尽管使用了不同的尺寸标准,膜囊泡范围通常为50~1000 nm,而外泌体的直径则为30~150 nm^[1,8]^。总体而言,EVs包含从30到1000 nm不等的各种大小的囊泡,且不同类型的囊泡在尺寸分布上存在重叠。

前列腺癌(prostate cancer, PCa)是发达国家中最常见的癌症之一,并且是导致男性癌症相关死亡的第二大原因。然而,现有的PCa诊断手段仍不够理想^[9⇓⇓-12]^。当前的筛查方法包括血清前列腺特异性抗原(prostate-specific antigen, PSA)检测、直肠指检(digital rectal examination, DRE),对于怀疑存在PCa的患者,通常建议使用磁共振成像或经直肠超声进行评估。然而,PSA检测的阳性预测值仅为25%~40%,并且在PSA血清水平介于4.0到10.0 ng/mL的男性中,约65%~70%最终被诊断为前列腺癌阴性^[13]^。PSA检测和DRE的特异性较低,导致了大量不必要的前列腺活检。因此,亟需更快速、可靠的生物标志物以提升PCa诊断的准确性。鉴于前列腺与尿道的解剖位置紧密相连,尿液中可检测到前列腺或其癌变分泌物,这使尿液成为PCa生物标志物液体活检的潜在来源,并在近年来得到了广泛研究。

尿液由于蛋白质浓度较低、盐分存在以及蛋白质表达的巨大动态范围,成为一种极具复杂性的液体介质,使得基于蛋白质的生物标志物发现面临巨大挑战^[14]^。尿液中的EVs通过富集蛋白质,有效克服了这些问题,同时减少了白蛋白和尿调节素等高丰度蛋白质的干扰。此外,EVs的分离过程还能显著降低样品中的盐浓度。尤其值得关注的是,EVs富含前列腺源性物质,能够从尿液中提取,且通常包含外泌体、微泡、蛋白质聚集体和大蛋白质复合物等颗粒^[15]^。因此,EVs在基于液体活检的PCa诊断生物标志物的发现中展现出巨大的潜力。目前,超速离心(ultracentrifugation, UC)方法被认为是外泌体分离的“金标准”,但超速离心存在回收率和通量较低、依赖昂贵的仪器等不足,其他方法如聚合物沉淀法、尺寸排阻色谱法、免疫亲和等也被用于EVs的分离,但这些方法仍在富集效率、灵敏度和分离通量等方面存在较大的局限性^[16,17]^。我们开发了一种基于3种抗体的亲和磁珠系统(extracellular vesicles isolated efficiently, naturally, and totally, EVlent),通过与EVs表面的蛋白质相互作用,实现了高效的EVs亲和富集^[18]^。相比超速离心,该方法不仅提高了富集效率,还因其具有磁性,可与自动化设备结合,实现样品的高通量处理,为大规模临床队列分析提供了强有力的工具。

在本研究中,我们使用EVlent对尿液来源的EVs进行富集,并对前列腺癌患者样本进行了蛋白质组学分析。EVlent通过表面修饰3种抗体(CD9、CD63、CD81),能够特异性识别并结合EVs表面蛋白质。富集的EVs通过免疫印迹(Western blotting, WB)分析、透射电子显微镜(TEM)和纳米颗粒追踪分析(NTA)验证了富集效率及其完整性。随后,对前列腺癌患者和健康对照组的临床样本进行了无标记定量蛋白质组学分析,并通过生物信息学筛选出若干潜在的前列腺癌生物标志物,为液体活检提供了一种新的诊断方法和手段。

## 1 实验部分

### 1.1 仪器、试剂与材料

nano-Easy 1200液相系统和QE HF-X质谱仪(Thermo Scientific, USA);冷冻浓缩仪(Labconco, USA);ZetaView纳米颗粒追踪分析仪(Particle Metrix, Germany), Tanon 5200图像分析仪(Tanon, China),高速冷冻离心机(Eppendorf, Germany), HITACHI HT-7800透射电子显微镜(Hitachi, Japan);旋转混合仪(海门市其林贝尔仪器制造公司。

磷酸盐缓冲液(phosphate buffered saline, 1×PBS)、水(质谱纯)、乙腈(ACN)、甲醇购自Thermo Scientific (USA);脱氧胆酸钠、月桂酰肌氨酸钠、三(2-羧乙基)膦、2-氯乙酰胺、三乙胺(triethylamine, TEA)、三乙胺碳酸氢盐和TritonX-100、ProClin 300、聚偏二氟乙烯膜购自Sigma(USA);CD9抗体购自CST公司,TSG101和Calnexin抗体购自Abcam公司,HSP70抗体购自Proteintech公司,磷钨酸购自国药集团化学试剂有限公司,胰蛋白酶和Lyc-C酶购自南京逸微健华生物科技有限公司,BCA蛋白测量试剂盒(P0010)购自碧云天生物技术有限公司,Tris缓冲盐Tween洗涤缓冲液(Tris Buffered Saline with Tween-20,1×TBST)、蔗糖和牛血清白蛋白(bovine serum albumin,BSA)购自Biosharp公司。其他试剂为实验室常用试剂。

### 1.2 样本采集

研究经江苏省人民医院医学伦理委员批准,批准号为2021-SR-167。参与者均在清晨空腹状态下采集尿液。尿液样本采集后,2500 g离心两次,每次10 min(以去除细胞碎片和大的凋亡小体),离心后上清液保存于-80 ℃备用。

### 1.3 EVlent磁珠的制备

根据我们之前报道的方法制备EVlent磁珠^[18]^。首先取10 mg羧基磁珠,用包被液(0.1 mol/L 2-吗啉乙磺酸)清洗3次。之后,将磁珠与0.5 mL 1-(3-二甲基氨基丙基)-3-乙基碳二亚胺(1-(3-dimethylaminopropyl)-3-ethylcarbodiimide, EDC, 10 mg/mL)和0.5 mL *N*-羟基丁二酰亚胺(*N*-hydroxy succinimide, NHS, 10 mg/mL)溶液混合,在搅拌器上充分混匀后,反应2 h以活化磁珠。活化后,再用包被液清洗3次。接着,将适量的抗CD9、CD63和CD81抗体加入到1 mL含10 mg重悬磁珠的溶液中,在室温下混匀反应6 h进行抗体偶联。偶联完成后,用封闭液(1×PBS, 含1%BSA 和 2.5%蔗糖)对磁珠清洗3次,然后加入1 mL封闭液,在室温下搅拌12 h进行封闭处理。最后,通过磁分离去除封闭液,加入1 mL磁珠保存液(1×TBST 和 1% ProClin 300 ),得到10 mg/mL的功能化EVlent磁珠。

### 1.4 EVlent富集尿液细胞外囊泡

取1 mL尿液样本,加入20 μL EVlent磁珠,室温孵育1 h,用磁力分离器去除上清液,用1 mL上样缓冲液(0.1% Triton-100)洗涤一次,用1 mL PBS溶液洗涤两次,最后用200 μL洗脱缓冲液(100 mmol/L TEA溶液)洗脱EVlent磁珠两次,得到EVs,收集的EVs洗脱液经冷冻干燥后保存在-80 ℃。

### 1.5 WB分析

采用十二烷基硫酸钠-聚丙烯酰胺凝胶电泳(SDS-PAGE)检测尿液中EVs的存在。将上述的EVs冻干品用40 μL PTS裂解液涡旋混匀充分后,在95 ℃水浴锅中煮10 min,冷却后离心,取20 μL进行蛋白质WB分析。一抗采用常见的EVs蛋白质(CD9、TSG101、HSP7、Calnexin)来验证,二抗采用辣根过氧化物酶连接的相应抗体,通过化学发光增强试剂盒和Tanon 5200图像分析仪进行WB分析。

### 1.6 TEM检测

将10 μL的EVs溶液滴入200目镀碳铜网中,自然干燥后,将样品与2%磷钨酸溶液(pH=7.0)在室温下孵育2~3 min以进行阴性染色。之后对EVs进行了透射电子显微镜成像。

### 1.7 NTA分析

通过NTA技术测定胞外囊泡粒径分布。首先将从1 mL尿液样本中富集到的胞外囊泡洗脱液用PBS稀释至10 mL。然后,每次NTA分析使用1 mL胞外囊泡PBS溶液。采用ZetaView纳米颗粒追踪分析仪进行测定,用100 nm聚苯乙烯颗粒用纯水稀释25万倍进行校准。仪器最小亮度设置为20,灵敏度设置为70,快门设置为100。

### 1.8 蛋白质组学分析

样品前处理 将冻干的EVs用裂解液进行处理,得到蛋白质裂解物。使用BCA试剂盒测定蛋白质浓度,按照酶与蛋白质质量比1∶100加入Lys-C酶,37 ℃酶解3 h。随后,按照1∶50的质量比加入胰蛋白酶,37 ℃继续酶解16 h。酶解后,通过C18柱进行脱盐处理,样品随后用于液相色谱-质谱分析。

色谱条件 C18色谱柱(20 cm×75 μm, 2.2 μm; Michrom Bioresources);柱温为40 ℃;流动相A相为0.1%甲酸水溶液,B相为80%乙腈水溶液(含0.1%甲酸),梯度程序如下:0~1 min, 5%B~10%B;1~52 min,10%B~32%B;52~53 min 32%B~95%B;53~60 min, 95%B。流速为300 nL/min。上样体积为1 μL。

质谱条件 离子源温度为320 ℃;正离子扫描模式;喷雾电压为2.1 kV;漏斗RF(Radio Frequency)水平为40。碰撞能量为27。使用数据非依赖性采集(data independent acquisition,DIA)模式进行扫描,扫描范围*m/z* 400~1200,分辨率为120000,隔离窗口为*m/z* 16,采集的数据通过Spectronaut 18软件进行检索。将酶设置为胰蛋白酶/P,最多漏切2个位点。选择氨基甲酰甲基化(+57.0214 Da)作为固定修饰,选择甲硫氨酸氧化(+15.9949 Da)和肽段N端乙酰化(+42.011 Da)作为可变修饰。肽段和蛋白质分别设定了1%的错误发现率作为临界值。

### 1.9 统计分析

利用Perseus软件对检索数据进行差异表达分析,所有蛋白质丰度都使用二进制对数进行转换,并将样本分为健康志愿者和前列腺癌患者两组。所有样本中丰度频率高于50%的蛋白质被认为是有效值。利用正态分布填补缺失值后对每个样品的丰度进行中位数归一化。然后对两组数据进行T检验(*p* < 0.05),最后,|log_2_ (Fold change)|>1为差异蛋白质。所有图片均使用GraphPad Prism (v8.0)、Origin2022 (v9.9)和R(4.2.3)绘制。

## 2 结果与讨论

EVs表面携带多种标志性蛋白质,如CD9、CD63和CD81等,这些蛋白质已被广泛用作EVs的生物标志物。尽管UC是分离EVs的常用方法,但其操作时间较长、回收率低,且往往伴随大量杂质。EVlent亲和磁珠通过抗原抗体相互作用来识别EVs表面的特异性蛋白质(CD9/CD63/CD81),能够快速、高效地从复杂的体液中分离出完整的EVs。图1展示了本研究的总体流程。首先,我们评估了EVlent在细胞外囊泡富集中的效率,随后针对前列腺癌患者尿液中的EVs进行LC-MS蛋白质组学分析,以筛选出潜在的前列腺癌相关蛋白质标志物。

**图1:**
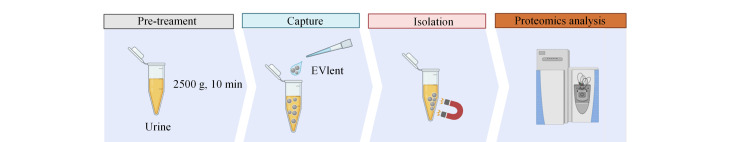
EVlent富集尿液中的EVs用于蛋白质组学研究

### 2.1 EVlent富集细胞外囊泡的表征

蛋白质免疫印迹法是用于EVs定性检测的常规手段,其中最常用的标志蛋白质包括CD81、CD63、CD9和TSG101,它们在EVs的形成与分泌过程中发挥关键作用。为验证EVlent方法的分离效率,我们从1 mL尿液中提取EVs,并以传统UC方法作为对照,比较EVs标志蛋白质CD9、TSG101和HSP70的表达水平。结果显示(图2a), EVlent分离的样品中,CD9、TSG101和HSP70的条带显著可见,而UC组的条带相对较弱。此外,无论是EVlent还是UC方法,均未检测到Calnexin的条带。这表明,EVlent富集方法在效果上明显优于UC。NTA结果证明分离的EVs大部分位于50~400 nm范围内,EVlent和UC分离的EVs含量分别为4.1×10^9^ particles/mL和1.8×10^9^ particles/mL(图2b)。从TEM可以看出(图2c、2d), EVs呈椭圆形或杯状囊泡结构。

**图2:**
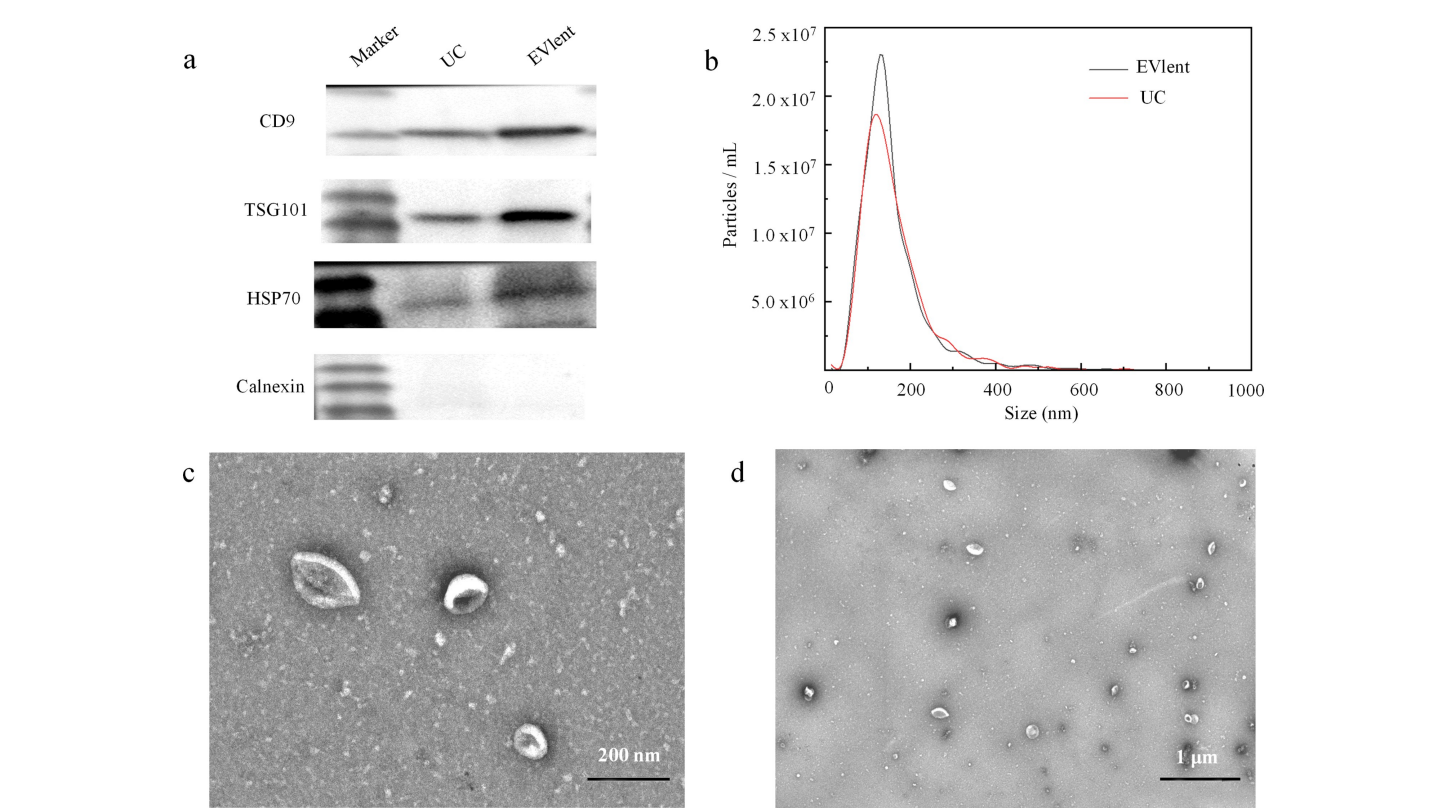
EVlent所富集EVs的相关表征

为更好地了解EVlent的性能,对该亲和磁珠的回收率进行了评估。将使用UC方法获得的纯EVs作为起始浓度(溶解在PBS中),分别利用EVlent亲和磁珠和UC方法对PBS中的EVs标准品进行捕获,捕获后进行蛋白免疫印迹分析。从图3a中可以看出,EVs标准品的CD9条带最深,UC方法CD9条带最弱。为了更清楚地比较回收率的高低,分别对它们的强度值进行统计,以EVs标准品的强度值作为1进行归一化处理。从图3b中可以看出,EVlent和UC方法的回收率分别是87.2%和30.3%。结果表明,EVlent亲和磁珠对EVs具有良好的回收率,其对EVs的回收效果远远高于UC方法。

**图3:**
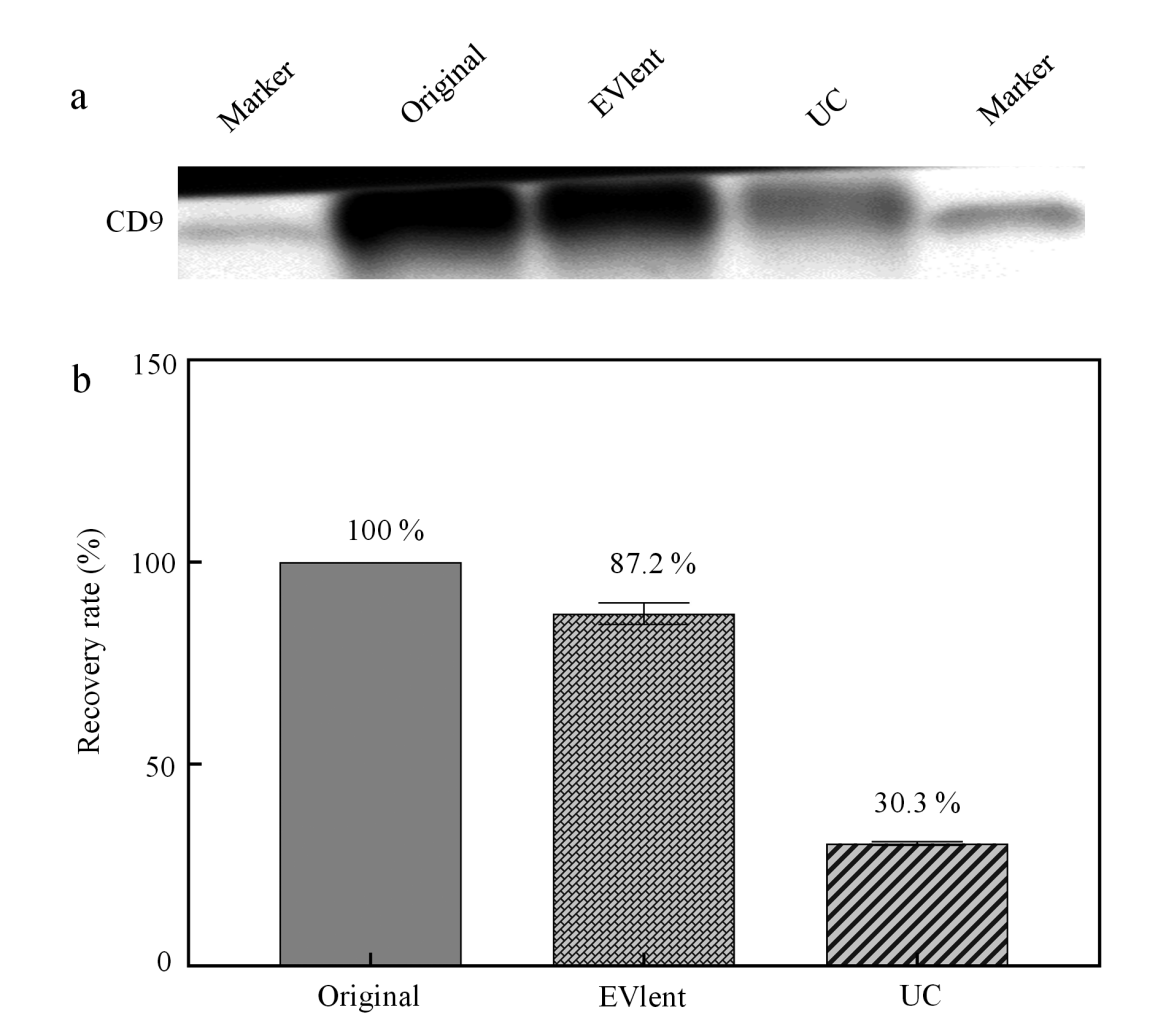
不同方法富集EVs的回收率(*n*=3)

### 2.2 尿液细胞外囊泡的蛋白质组学分析

为研究前列腺癌患者与健康志愿者之间EVs蛋白质组的差异,我们采用了非标记定量的方法。此外,将人工合成肽(iRT)加标到每个样品中并用作内标,以减少运行间差异并辅助肽段定量,在质谱运行中具有较好的一致性。原始数据已上传至科学数据银行,对应的数据CSTR编号为1253.11.sciencedb.sepu.00003(https://cstr.cn/31253.11.sciencedb.sepu.00003)。分析了15名前列腺癌患者(将5个样本混合为1个,共获得3个样本:PC1、PC2、PC3)和15名健康志愿者(同样将5个样本混合为1个,共获得3个样本:Control 1、Control 2、Control 3)尿液中的EVs。结果显示(图4a),健康志愿者组(Control 1~Control 3)尿液中的EVs平均鉴定出2039种蛋白质和14490条肽段,而前列腺癌患者组(PC1~PC3)中平均鉴定出1982种蛋白质和13100条肽段(具体见附表S1和附表S2, www.chrom-China.com),二者EVs中蛋白质和肽段的鉴定数量相近。

**图4:**
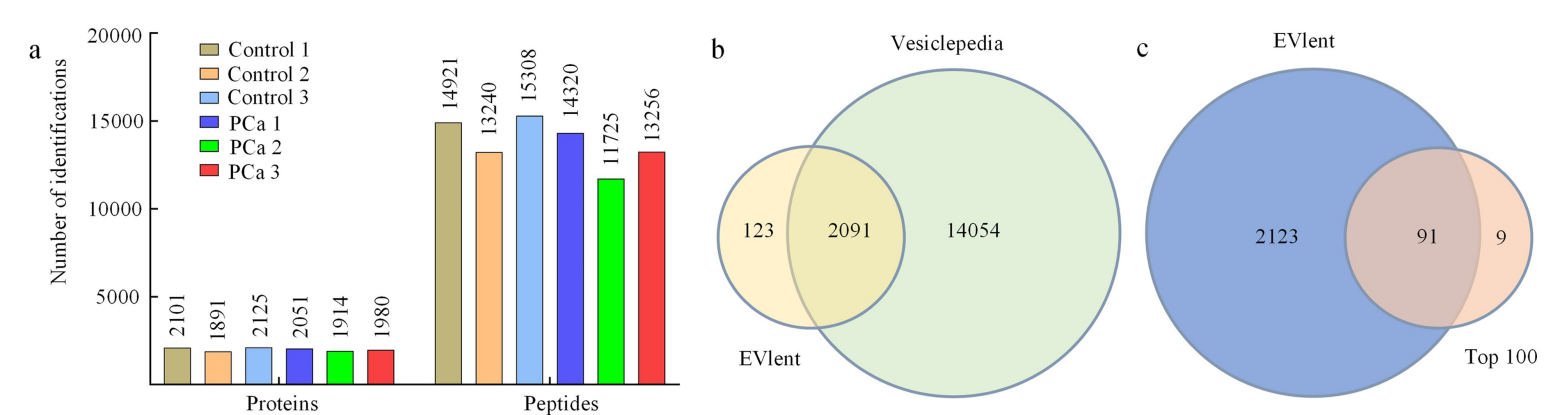
健康志愿者(Control 1~Control 3)与前列腺癌患者(PC1~PC3)的尿液细胞外囊泡蛋白质组学分析

将健康志愿者和前列腺癌患者鉴定到的尿液EV蛋白质取并集,一共鉴定到了2214个蛋白质,通过将鉴定到的EV蛋白质与Vesiclepedia数据库中包含的所有细胞外囊泡分子信息进行比较,结果显示84%的尿液EV蛋白质在该数据库中得到了确认(图4b)。此外,在Top 100 EV蛋白质中,我们鉴定到91种(图4c)。这些结果表明,EVlent对尿液EV富集表现出高选择性,为后续肿瘤标志物筛选的特异性提供了保障。

### 2.3 前列腺癌患者与健康志愿者尿液EVs差异表达分析

已经报道了很多种前列腺癌相关的潜在生物标志物,例如上皮细胞黏附分子(EPCAM)^[19]^。EPCAM是一种跨膜糖蛋白,在快速增殖的癌症中高度表达,在预防细胞间黏附、细胞信号传导、迁移、增殖和分化中起重要作用。在6个样本的质谱鉴定结果中,我们发现了相似的趋势,即前列腺癌患者中的EPCAM蛋白质表达量增加(具体见附表S1)。为了筛选出前列腺癌中更可信的潜在生物标志物,我们对两组数据进行了生物信息学分析。前列腺癌患者和健康志愿者的尿液EVs中具有明显丰度差异,蛋白质火山图分析如图5a所示,一共筛选到了178个差异蛋白,其中88个蛋白质的表达量上调,有90个蛋白质表达量下调(|log_2_ (Fold change)|>1, *p*<0.05)(具体见附表S3)。在上调蛋白质中,至少有11个蛋白质曾被报道与前列腺癌生物学特性相关,这11个蛋白质为聚糖结合蛋白(SDCBP)、醛酮还原酶1B10(AKR1B10)、Copine 3蛋白(CPNE3)、趋化因子配体14(CXCL14)、尿激酶型纤溶酶原激活剂(PLAU)、神经母细胞瘤RAS病毒致癌基因同系物(NRAS)、α1-微球蛋白/比库宁前体(AMBP)、血小板衍生生长因子A(PDGFA)、ATP酶Na^+^/K^+^转运亚基β1(ATP1B1)、基质金属蛋白酶3(MMP3)以及基质金属蛋白酶7(MMP7)等。通过*z*-score归一化之后,得到的热图则进一步更直观表现出这些全蛋白在病人组和对照组的表达差异性(图5b, *p* < 0.05)。随后,对这88个上调蛋白质进行了GO分析,结果如图4c所示,分类显示,这88个上调的全蛋白中的大部分属于细胞外泌体、细胞外间隙、细胞外区域等,并且与蛋白结合作用相关。

**图5:**
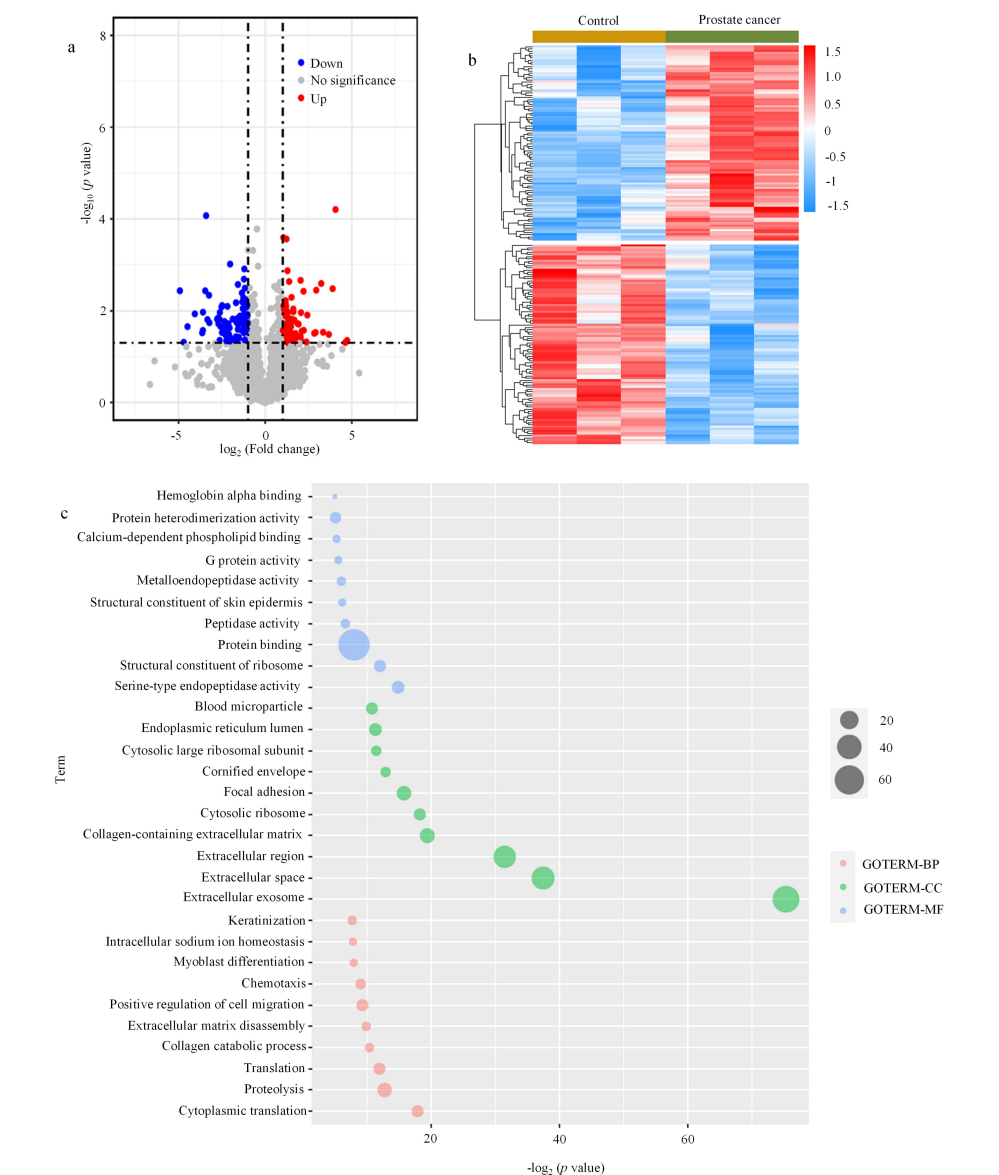
前列腺癌患者与健康志愿者尿液EVs差异表达蛋白质分析

为了进一步研究前列腺癌尿液EVs的生物标志物,我们对88种蛋白质进行了KEGG通路分析。结果显示(图6),这些蛋白质与前列腺癌、蛋白质消化吸收、补体和凝血级联以及癌症中的转录失调等通路相关。在前列腺癌通路中,我们富集了4种蛋白质:PLAU、PDGFA、MMP3和NRAS。我们重点对这4种蛋白质进行了深入研究。

**图6:**
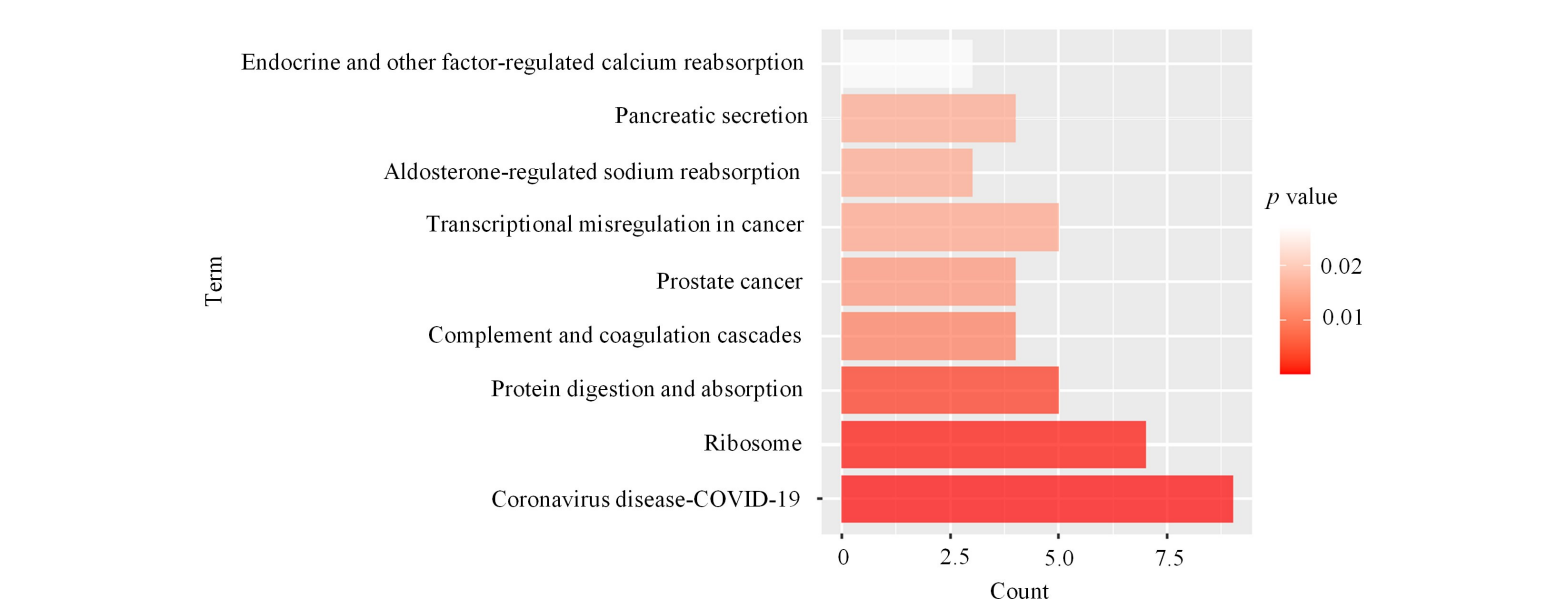
尿液EVs中上调蛋白质的KEGG分析

PLAU在癌症进展中发挥着重要作用。它通过蛋白质水解系统、细胞内信号传导和趋化因子的激活,促进细胞增殖、运动、黏附等多种生物学活动^[20]^。研究显示,PLAU的高表达与胰腺导管腺癌的侵袭性增强、基质评分升高及免疫抑制密切相关^[21]^。此外,PLAU还能够通过Src/ERK信号通路促进结直肠癌细胞的迁移、浸润和增殖^[22]^。近期,Nassir等^[23]^的研究表明,前列腺癌患者的PLAU水平显著高于良性前列腺增生患者(*p*<0.001),且在受试者工作特征曲线分析中显示出较高的曲线下面积。

PDGFA的高水平不仅与前列腺癌的发生、进展及骨生物学变化密切相关,长期以来还被认为与预后不良和转移密切相关。PDGFA促进细胞增殖和迁移,并被认为是前列腺癌进展中的成骨相关基因^[24,25]^。在癌变的前列腺组织中PDGFA蛋白上调,能够刺激多种类型的细胞生长、存活和迁移,并通过旁分泌和自分泌途径加速前列腺癌的发生与进展。此外,Gabriel等^[26]^的研究显示,与无病组织相比,PDGFA在前列腺肿瘤及癌旁完整组织中的表达显著升高,提示其可能在区域性前列腺癌的发展中起作用。

基质金属蛋白酶(MMPs)是一类由基质细胞和肿瘤细胞分泌的蛋白酶家族,其在多种癌症中均呈现升高趋势,并与患者的生存率负相关^[27]^。已有研究表明,MMP1、3、7和9在前列腺癌骨转移过程中发挥重要作用,其中MMP3的多态性与前列腺癌风险增加相关,特别是在骨转移患者中,MMP3水平显著升高^[28]^。Frieling等^[29]^的研究进一步证实,前列腺癌细胞来源的MMP3控制内源性细胞增殖和外源性血管生成,选择性抑制MMP3或靶向MMP的代谢产物,可能成为治疗前列腺癌骨转移的新策略。NRAS基因属于RAS致癌基因家族,是肿瘤发生中的关键基因之一,在细胞分裂、分化及凋亡过程中发挥重要调控作用。致病性突变导致*NRAS*基因编码的NRAS蛋白持续活化,进而引发细胞的异常增殖,最终形成肿瘤^[30]^。Meng等^[31]^通过RT-PCR评估了前列腺癌组织中NRAS的表达情况,结果显示,与正常组织相比,NRAS在肿瘤组织中的表达显著上调。进一步的研究表明,NRAS表达的增加与部分患者的激素难治性前列腺癌相关,且NRAS在细胞膜上的表达增加与较短的复发时间(*p*=0.01)及较低的疾病特异性生存期(*p*=0.008)相关。此外,NRAS膜表达增加的患者在复发时PSA水平较低^[32]^。

## 3 结论

本研究首次应用EVlent亲和磁珠对尿液样本中的EVs进行富集和多种表征,开发了一种高效的尿液EVs富集策略。通过该策略,对前列腺癌患者尿液中的EVs进行了富集和蛋白质组学分析,筛选出88种上调蛋白质和90种下调蛋白质。随后,对上调蛋白质进行了KEGG通路分析,鉴定出与前列腺癌相关的4种潜在生物标志物:PLAU、PDGFA、MMP3和NRAS。这些蛋白质可能在前列腺癌的诊断和预后评估中具有重要的临床应用价值,为基于EVs的肿瘤液体活检提供了重要的科学依据。
